# Expert vs. novice differences in the detection of relevant information during a chess game: evidence from eye movements

**DOI:** 10.3389/fpsyg.2014.00941

**Published:** 2014-08-25

**Authors:** Heather Sheridan, Eyal M. Reingold

**Affiliations:** ^1^School of Psychology, University of SouthamptonUK; ^2^Department of Psychology, University of Toronto at MississaugaMississauga, ON, Canada

**Keywords:** visual expertise, expert performance, chess, eye movements, attention, relevancy

## Abstract

The present study explored the ability of expert and novice chess players to rapidly distinguish between regions of a chessboard that were relevant to the best move on the board, and regions of the board that were irrelevant. Accordingly, we monitored the eye movements of expert and novice chess players, while they selected white's best move for a variety of chess problems. To manipulate relevancy, we constructed two different versions of each chess problem in the experiment, and we counterbalanced these versions across participants. These two versions of each problem were identical except that a single piece was changed from a bishop to a knight. This subtle change reversed the relevancy map of the board, such that regions that were relevant in one version of the board were now irrelevant (and vice versa). Using this paradigm, we demonstrated that both the experts and novices spent more time fixating the relevant relative to the irrelevant regions of the board. However, the experts were faster at detecting relevant information than the novices, as shown by the finding that experts (but not novices) were able to distinguish between relevant and irrelevant information during the early part of the trial. These findings further demonstrate the domain-related perceptual processing advantage of chess experts, using an experimental paradigm that allowed us to manipulate relevancy under tightly controlled conditions.

The remarkable perceptual skill of experts is exemplified by expert radiologists who can detect abnormalities in chest X-rays that were briefly presented for only 200 ms (Kundel and Nodine, 1975), and by chess experts who can memorize chessboards that were presented for only a few seconds (De Groot, 1946, 1965; Chase and Simon, 1973a,b). Furthermore, while examining visual displays that require multiple eye fixations for encoding, experts are adept at rapidly focusing their attention on relevant areas, such that radiologists can rapidly fixate on abnormalities (Kundel et al., 2008), and chess experts can rapidly fixate on the best move on a chessboard (Charness et al., 2001). Given that this fast extraction of relevant information is a key component of skilled performance in many different domains of expertise (for a review see Reingold and Sheridan, 2011), the goal of the present experiment is to further explore expert/novice differences in the detection of relevant information during a chess game. Accordingly, we will begin by briefly reviewing prior work on perceptual skill in the domain of chess, and we will then describe the present study's paradigm and rationale.

Of relevance to the present study, there is a long history of studying the perceptual component of expertise in the domain of chess (for reviews, see Charness, 1992; De Groot and Gobet, 1996; Reingold and Charness, 2005; Gobet and Charness, 2006; Reingold and Sheridan, 2011). The chess domain provides numerous methodological advantages, such as well-segmented visual stimuli for eye movement interest areas, and an official rating system for objectively quantifying levels of expertise (Elo, 1965, 1986). Capitalizing on these methodological advantages, chess expertise has been linked to numerous perceptual processing advantages, including superior memory performance for briefly presented chessboards (De Groot, 1946, 1965; Chase and Simon, 1973a,b), the ability to process chess configurations automatically and in parallel (Reingold et al., 2001b), and a larger *visual span* such that experts process larger configurations of pieces than novices (Reingold et al., 2001a). Consistent with these behavioral and eye movement findings, neuroimaging work has shown expert/novice differences in brain activation in regions associated with object and pattern recognition (Bilalić et al., 2010a, 2011a,b, 2012). Taken together, the above findings collectively support the view that perceptual skill is a key aspect of expertise in chess (De Groot, 1946, 1965; Chase and Simon, 1973a,b) as well as in other domains of visual expertise (for a review see Reingold and Sheridan, 2011).

To provide a theoretical account of the perceptual skill shown by chess experts, Chase and Simon (1973a,b) proposed that through thousands of hours of practice chess experts acquire memories for a large number of “chunks,” which consist of groups of chess pieces, and these chunks are supplemented by larger memory structures called templates (Gobet and Simon, 1996, 2000). Such memory structures facilitate performance by enabling chess players to rapidly retrieve useful information, such as advantageous strategies and moves. Thus, Chase and Simon (1973a,b) argued that chess experts use their memory for chess-configurations to constrain their search for a move to the most promising candidates, rather than performing a slow and exhaustive search of all of the possible moves on the board. This theoretical perspective echoes earlier arguments by De Groot (1946, 1965) that chess expertise stems from advantages in memory and perception, rather than from a greater breadth and depth of search during problem solving.

Consistent with this hypothesis that chess experts rely on their memory for chess configurations to efficiently guide their search for the best move, the eye movements of chess experts reveal that they can rapidly fixate on information that is relevant to the best move on the board (Tikhomirov and Poznyanskaya, 1966; Simon and Barenfeld, 1969; Charness et al., 2001; Reingold and Charness, 2005). For example, to examine the impact of relevancy on eye movements, Charness et al. (2001) monitored the eye movements of expert players (mean Elo rating = 2238) and intermediate players (mean Elo = 1786) while they selected white's best move in a series of chess problems (henceforth, the *choose-a-move task*). Compared to intermediate players, the experts produced a greater proportion of fixations on pieces that were relevant to the best move, and this advantage of the experts emerged as early as the first five fixations in the trial (for a discussion of similar findings, Tikhomirov and Poznyanskaya, 1966; Simon and Barenfeld, 1969). As a follow-up to Charness et al. (2001), Reingold and Charness (2005) analyzed the first 10 s of choose-a-move trials to demonstrate that experts rapidly completed a perceptual encoding phase (characterized by shorter fixations) and then shifted to a subsequent problem-solving stage (characterized by longer fixations). In marked contrast, the intermediates continued to display shorter fixations throughout the 10-s period, which indicates that they needed more time to complete the perceptual encoding phase. Taken together, these studies indicate that chess experts are more efficient at encoding chess configurations during a choose-a-move task.

Beyond the choose-a-move task, there is further evidence that experts are better than novices at rapidly encoding relevant chess configurations. For example, in a memorization task, De Groot and Gobet (1996) demonstrated that the number and total duration of fixations on chess pieces was at least partially correlated with the relevance of these pieces to the position, and the magnitude of this correlation increased as a function of skill. Moreover, using a chess-related visual search task that required participants to search for relevant pieces on a chessboard, Bilalić et al. (2010a) revealed that chess experts (but not novices) were able to rapidly and exclusively fixate on task-relevant rather than irrelevant features. Finally, Bilalić et al. (2012) examined relevancy effects in a threat detection task, in which experts and novices had to examine chessboards to determine the number of black pieces that were attacking white pieces. The experts displayed a higher percentage of fixations on relevant objects (i.e., the pieces that formed a threat relationship) relative to novices, and this difference between experts and novices emerged as early as the first three seconds in the trial. Based on these results, Bilalić et al. (2012) concluded that the “experts' advantage lies in the ability to immediately focus on relevant objects and relations between them in the environment and ignore the irrelevant ones.”

Building on this prior work, the present study introduces a new paradigm for studying relevancy effects in chess. Similar to prior work (Charness et al., 2001; Reingold and Charness, 2005; Bilalić et al., 2008a,b, 2010b; Sheridan and Reingold, 2013), the present paradigm monitored the eye movements of chess players during a choose-a-move-task, which is an ecologically valid task given that it resembles the challenges confronting chess players during a chess match. To provide a well-controlled manipulation of relevancy, the present paradigm employs two counterbalanced versions of each chess problem, which differed by a single piece such that a bishop was changed to a knight (see Appendix A in Supplementary material for examples). This subtle change reversed the relevancy map of the board, such that the regions that were relevant to the best move in one version are irrelevant in the other version, and vice versa. Thus, the present paradigm extends prior work by employing large relevant and irrelevant regions of interest that were well-matched on a variety of characteristics (e.g., number of squares, location, etc.).

Our rationale for using this paradigm was to contrast the time-course of relevancy effects in both novice and expert players. Thus, we asked strong expert players (average Elo = 2223) and novices (unrated club players) to solve a series of problems that were designed to be simple enough that both the novice and expert players could frequently detect the best move on the board. In light of past findings concerning the perceptual encoding advantage of experts, we expected that the expert's eye movements would reveal an earlier differentiation between relevant and irrelevant regions. Such a finding would provide additional support for the importance of perceptual processing in chess skill, using a new paradigm that afforded a number of methodological advantages.

## Methods

### Participants

Forty-one chess players (17 experts and 24 novices) were recruited from online chess forums and from local chess clubs in Toronto and Mississauga (Canada). The mean age was 30 (*SD* = 14.2) in the expert group, and 27 (*SD* = 10.0) in the novice group. There was one female player in the expert group, and there were five female players in the novice group. For the expert players, the average CFC (Canadian Chess Federation) rating was 2223 (range = 1876–2580). All of the novice players were unrated club players who were familiar with the rules of chess, but had never participated in a rated chess tournament. All of the participants had normal or corrected-to-normal vision.

### Materials and design

There were eight experimental problems (See Appendix A in Supplementary material for the complete list of problems). To manipulate relevancy, we constructed two versions of each of the problems, and these two versions were identical except that a single piece was changed from a bishop to a knight. As shown in Appendix A in Supplementary material, this subtle change reversed the relevancy map of the board, such that regions that were relevant to the best move in one version were no longer relevant (and vice versa). Similar to Charness et al. (2001), relevancy was determined by asking an international master who did not participate in the study to classify the squares on the board as either relevant or irrelevant (see also De Groot and Gobet, 1996). For example, in the first version of Problem #3 (see panel 3a in Appendix A in Supplementary material), the best move on the board is “Rook to a8 (checkmate),” and the squares associated with this move are located on the left side of the board (see relevant region in orange), whereas the other side of the board contains squares that are irrelevant to the best move (see irrelevant region in blue). In contrast, in the second version of the problem (see panel 3b in Appendix A in Supplementary material) we changed a single piece from a bishop to a knight (see piece inside the dotted lines), such that the best move on the board became “Knight to f4,” and the relevant and irrelevant regions were reversed. The relevant and irrelevant regions were always located near the edge of the board, and never overlapped with the center of the board. The two versions of the problems were counterbalanced such that each player only saw one version of a given problem. The same order of problems was used for all players, and each chess player completed a total of eight experimental problems. It was always white's turn to move, and the problems incorporated a variety of solutions that ranged from checkmate to material gains to defensive tactics.

### Apparatus and procedure

Eye movements were measured with an SR Research EyeLink 1000 system with high spatial resolution and a sampling rate of 1000 Hz. The experiment was programmed and analyzed using SR Research Experiment Builder and Data Viewer software. Viewing was binocular, but only the right eye was monitored. A chin rest and forehead rest were used to minimize head movements. Following calibration, gaze-position error was less than 0.5°. The chess problems were presented using images (755 × 755 pixels) that were created using standard chess software (Chessbase 11). These images were displayed on a 21 in. ViewSonic monitor with a refresh rate of 150 Hz and a screen resolution of 1024 × 768 pixels. Participants were seated 60 cm from the monitor, and the width of one square on the chessboard equaled approximately 3.4 degrees of visual angle.

Prior to the experiment, the participants were instructed to choose white's best move as quickly and as accurately as possible, and they were told that they would be given a maximum of 3 min to respond to each problem. At the start of each trial, the participants were required to look at a fixation point in the center of the screen, prior to the presentation of the chessboard. The participants were asked to press a button as soon as they had made their decision, and they then reported their move verbally to the experimenter. If 3 min elapsed prior to the button press (this occurred on less than 1% of the experimental trials for the novices, and 0% of trials for the experts), then the chessboard was removed from the screen and the chess player was prompted to immediately provide their best answer.

## Results

Our main goal was to use eye movements to examine expert vs. novice differences in how attention was allocated to the relevant and irrelevant regions of the chessboard. However, prior to reporting the eye movement results, we will first report several global measures of performance (i.e., accuracy, reaction times) as a function of the chess player's level expertise (expert, novice).

To assess move quality, we asked an international chess master who did not participate in the study to rate the quality of each move on a scale from 1 to 10 (1 = a blunder, 10 = one of the best moves on the board), and we consulted the evaluation function from a chess engine (Houdini 2 Pro), which provides a score (expressed in pawn units) to quantify the change in White's positional advantage as a result of the move chosen. For both of these measures of accuracy, the experts showed superior performance than the novices (*df* = 39; all *t*s > 2.0, all *p*s < 0.05). Specifically, the average move quality rating was 9.5 (*SE* = 0.12) for the experts and 6.9 (*SE* = 0.26) for the novices, and the average chess engine score was 2.1 (*SE* = 0.17) for the experts and 1.5 (*SE* = 0.16) for the novices. Moreover, the experts selected the best move on the board (i.e., a move that received a rating of 10), on an average of 93 % of trials (*SE* = 2%), whereas the novices selected the best move on an average of 52% of trials (*SE* = 5%), and this expert/novice difference in accuracy was significant: *t*_(39)_ = 7.12, *p* < 0.001. In addition, there was a marginally significant trend toward faster reaction times for the experts (*M* = 28,946 ms; *SE*= 5441 ms) than for the novices (*M* = 41,854 ms; *SE* = 4748 ms), *t*_(39)_ = 1.78, *p* = 0.084. More interestingly, within the expert group there was a negative correlation between the mean reaction time of each player and their Elo rating (*r* = −0.617, *p* < 0.01), which indicates that increases in chess rating were associated with faster performance. Finally, the experts' (but not the novices') reaction times were significantly faster when the relevant region was on the right rather than the left side of the board, *t*_(16)_ = 2.16, *p* < 0.05 (for similar findings, see De Groot and Gobet, 1996).

**Figure 1:**
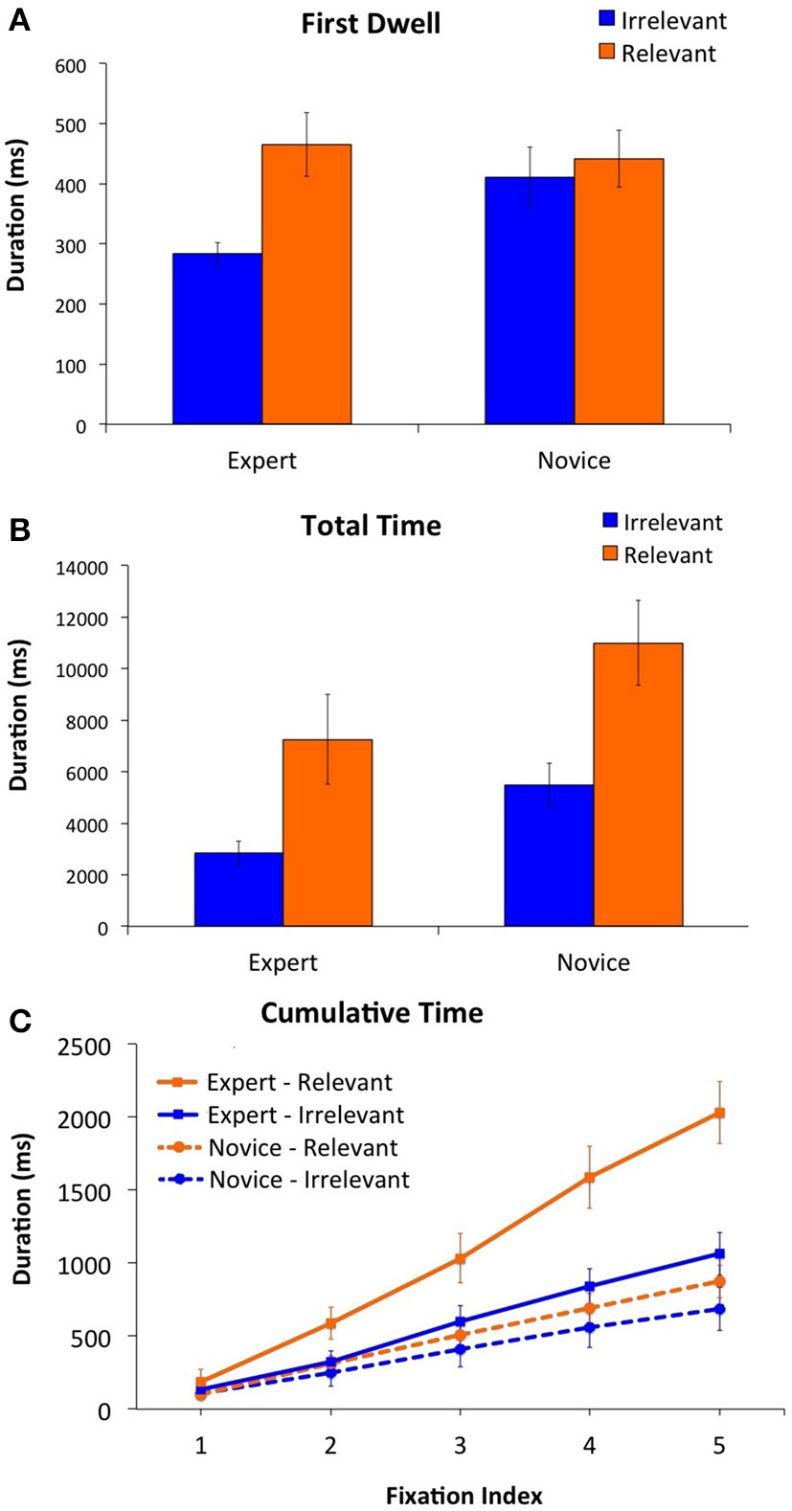
**The duration of the First Dwell (A) Total Time (B), and the Cumulative Time of the first five ordinal fixations in the trial (C) as a function of relevancy (relevant vs. irrelevant) and level of expertise (expert, novice)**.

Next, we analyzed eye movements to examine the extent to which the expert and novice chess players directed their attention toward the relevant and irrelevant regions of the board. For all of the eye movement analyses reported below, we analyzed correct trials only (i.e., trials that elicited a 10-rated move), to ensure that the experts and novices were matched for accuracy. Given our interest in the time-course of relevancy effects, we began our analysis by examining an early measure of processing (i.e., *first-dwell duration*, which is the duration of the first dwell on a given region, where a dwell is defined as one or more consecutive fixations on the target region, prior to the eyes moving to a different region of the board) as well as a later measure of processing (i.e., *total time*, which is the sum of the duration of all of the dwells on a region for the entire trial). To explore the pattern of results for each measure, we examined 2 × 2 ANOVAs that included relevancy (relevant, irrelevant) as a within-subjects factor, and expertise (expert, novice) as a between-subjects factor. For both the first-dwell and total time measures, there was a main effect of relevancy (i.e., longer dwells on relevant than irrelevant regions), all *F*s > 8, all *ps* < 0.01, and a main effect of expertise (i.e., longer dwells for novices than experts), all *F*s > 60, all *ps* < 0.001. More importantly, there was a significant interaction for the first-dwell measure [*F*_(1, 39)_ = 4.38, *p* < 0.05], but not for the total time measure (*F* < 1). As can be seen from Figures 1A,B, this interesting dissociation between these two measures occurred because the first dwell measure produced significant relevancy effects for the experts [*t*_(16)_ = 3.51, *p* < 0.01] but not for the novices (*t* < 1), whereas the total time measure produced relevancy effects for both groups (all *t*s > 2, all *p*s < 0.05). Interestingly, the interaction between expertise and relevancy for the first-dwell measure was solely due to an increase in the number of fixations for relevant compared to irrelevant regions for the experts [relevant: *M* = 2.19, *SE* = 0.21, irrelevant: *M* = 1.39, *SE* = 0.07, *t*_(16)_ = 3.95, *p* < 0.01] but not for the novices (relevant: *M* = 2.05, *SE* = 0.16, irrelevant: *M* =1.98, *SE* = 0.23, *t* < 1) as shown by a significant interaction, *F*_(1, 39)_ = 8.28, *p* < 0.01. In contrast, the mean fixation duration for the first dwell did not vary as a function of relevancy or expertise (all *Fs < 1*).

The above first-dwell findings imply that experts are faster than the novices at detecting relevant information. To further explore this effect, we analyzed the first five fixations of the trial to quantify the amount of time that experts and novices spent fixating the relevant and irrelevant regions at the start of the trial (a similar analysis of the first five fixations was conducted by Charness et al., 2001). Specifically, for each fixation position ranging from one to five (fixation position one corresponded to the fixation which began following the initial saccade in the trial), we calculated the cumulative sum of all of the fixations on the relevant and irrelevant regions up to and including the current fixation position. This analysis was conducted separately for each participant and each condition (i.e., relevant vs. irrelevant), and then averaged across participants to produce the figure shown in Figure 1C. As can be seen from this figure, the experts showed stronger and earlier relevancy effects than the novices. This pattern of results was reflected in a three-way interaction, *F*_(4,36)_ = 2.74, *p* < 0.05 when we examined a 5 × 2 × 2 ANOVA that included fixation position (1,2,3,4,5) and relevancy (relevant, irrelevant) as within-subjects variables, and expertise (expert, novice) as a between-subjects variable. Consistent with this three-way interaction, the experts showed a significant relevancy effect [*F*_(1, 16)_ = 6.64, *p* < 0.05] that became stronger over time as shown by a relevancy by time interaction [*F*_(4, 13)_ = 6.67, *p* < 0.01], whereas the novices did not show a relevancy effect or an interaction (all *F*s < 1).

Taken together, the first-dwell findings and the cumulative time analyses indicate that experts are faster at detecting relevant information than novices, which supports the notion that chess expertise reflects an advantage in encoding chess-related visual configurations (De Groot, 1946, 1965; Chase and Simon, 1973a,b; De Groot and Gobet, 1996; Charness et al., 2001; Reingold et al., 2001a,b; Bilalić et al., 2010a, 2011a,b, 2012; for reviews see Reingold and Charness, 2005; Reingold and Sheridan, 2011). During the present study, this perceptual processing advantage enabled skilled players to rapidly focus on chess configurations that were relevant to the best move on the board.

## Discussion

The present experiment examined the time-course and magnitude of relevancy effects on expert and novice chess players' eye movements during a choose-a-move task. Our most important finding was that the eye movements of the experts, but not the novices, revealed a rapid differentiation between regions of the chess board that were relevant vs. irrelevant to the best move on the board. Specifically, the experts, but not the novices, spent more time looking at relevant than irrelevant regions during the early part of the trial (i.e., during the first-dwell on a region, and during the first five fixations of the trial), whereas both the experts and novices showed strong relevancy effects later on in the trial. Importantly, these findings were obtained using an experimental paradigm that provided a well-controlled manipulation of relevancy, such that the relevant and irrelevant regions of the board were counterbalanced across participants (see Appendix A in Supplementary material).

Similar to the present findings, prior studies have also shown enhanced relevancy detection by chess experts (Tikhomirov and Poznyanskaya, 1966; Simon and Barenfeld, 1969; De Groot and Gobet, 1996; Charness et al., 2001; Bilalić et al., 2010a, 2012). Moreover, this relevancy detection advantage is a particular instance of the perceptual encoding advantage that has been shown by chess experts in a variety of tasks employing domain-related stimuli (De Groot, 1946, 1965; Chase and Simon, 1973a,b; De Groot and Gobet, 1996; Charness et al., 2001; Reingold et al., 2001a,b; Bilalić et al., 2010a, 2011a,b, 2012; for reviews see Reingold and Charness, 2005; Reingold and Sheridan, 2011). To explain this perceptual encoding advantage, Chase and Simon (1973a,b) proposed that chess expertise develops due to extensive practice with domain-related visual-configurations. Over the course of thousands of hours of practice, chess experts store memories for configurations of chess pieces in memory in the form of chunks (Chase and Simon, 1973a,b), which are supplemented by larger memory structures called templates (Gobet and Simon, 1996, 2000). These memory structures lead to a perceptual encoding advantage such that chess experts are able to process chess stimuli in terms of larger configurations of pieces, rather than individual features. Consequently, chess players are able to use their memory for chess configurations to guide their search for the best move on the board, rather than exhaustively searching all possible moves. This theoretical account is consistent with the present study's finding that the chess experts were able to rapidly focus on information that was relevant to the best move on the board.

Beyond the chess domain, the present findings are also consistent with findings from other domains concerning the importance of perceptual processing during skilled performance. As reviewed by Reingold and Sheridan (2011), experts in many domains of expertise have been shown to efficiently process domain-related material in terms of larger configurations, as shown by findings that radiologists can rapidly fixate abnormalities in less than a second (Kundel et al., 2008). Moreover, this key role of perceptual processing in expertise coincides with other evidence for perceptual specificity effects in memory and learning (for reviews, see Levy, 1993; Roediger and McDermott, 1993; Reingold, 2002), such as recent findings that eye fixation times are shorter for words that were read twice in the same typography (i.e., font) rather than in two different typographies (Sheridan and Reingold, 2012a,b), and findings that chess experts perform better when viewing familiar chess symbols compared to a condition in which letters (i.e., B = Bishop, P = Pawn, etc.) were shown instead of the symbols (Reingold et al., 2001a).

More generally, the relevancy effects from the present study add to the growing evidence that higher level cognitive processes can rapidly influence eye movement control (e.g., the duration and location of fixations) on a variety of tasks. In fact, ever since the seminal eye tracking work by Yarbus (1967), it has been well-known that our eye movements are biased toward aspects of a visual image that are relevant to our current goals, and away from areas that are irrelevant. As further evidence for the role of higher cognitive processing in guiding eye movements, participants in visual search studies spend more time fixating on distractors that are related (e.g., visually similar) rather than unrelated to the target (e.g., Findlay and Gilchrist, 1998; Reingold and Glaholt, 2014), participants in face perception tasks preferentially look at relevant features, such as the eyes (e.g., Henderson et al., 2005), participants in scene perception tasks spend more time fixating information that is task-relevant rather than irrelevant (Glaholt and Reingold, 2012), and the eye movements of skilled readers reveal rapid effects of a variety of higher-level lexical, linguistic and cognitive variables (e.g., Rayner, 1998, 2009; Staub et al., 2010; Staub, 2011; Reingold et al., 2012; Sheridan and Reingold, 2012c,d). Taken together, these findings lend support to models of eye movement control that predict a strong *eye-mind link*, such that ongoing cognitive processing can have a rapid effect on lower-level perceptual and oculomotor processing (for recent reviews, see Reingold et al., 2012, in press). Moreover, these findings underscore that skilled performance reflects a complex inter-play of perceptual and cognitive processing, and future work can examine the extent to which similar findings from multiple domains are reflective of common underlying mechanisms.

Finally, a key contribution of the present study is that we introduced a new experimental paradigm to provide a carefully controlled manipulation of relevancy. As shown in Appendix A in Supplementary material, we created two counterbalanced versions of each chess problem that differed by a single piece, and the regions that were relevant in one version were irrelevant in the other version (and vice versa). Given that the relevant and irrelevant regions of the board were well-matched, we can conclude that the relevancy effects in the experiment were solely due to the relevancy of a given region to the best move, and not to some other confound (e.g., differences in visual saliency, location on the board, number of pieces in the region, etc.). The present paradigm could be used in the future to investigate additional topics concerning the impact of relevancy on a variety of aspects of chess expertise.

### Conflict of interest statement

The authors declare that the research was conducted in the absence of any commercial or financial relationships that could be construed as a potential conflict of interest.
