# Supplementary material for: Expert vs. novice differences in the detection of relevant information during a chess game: evidence from eye movements
Source: Front Psychol. 2014 Aug 25;5:941. doi: 10.3389/fpsyg.2014.00941 (PMC4142462; doi:10.3389/fpsyg.2014.00941)
Supplement: Supplementary file 1 [file DataSheet1.DOCX]

**Appendix A**

For the chess problems shown here, the relevant regions are highlighted in orange, and the irrelevant regions are highlighted in blue (note that the coloured regions are shown here for illustration purposes only, and were not shown to participants). For each pair of problems, the regions that were relevant in one version of the problem were irrelevant in the other version, and vice versa. The two versions of the problems differed by a single piece (indicated here with a dotted line), which changed from a bishop to a knight (or vice versa). It was always white’s turn to move, and the best move(s) are shown below each of the problems.


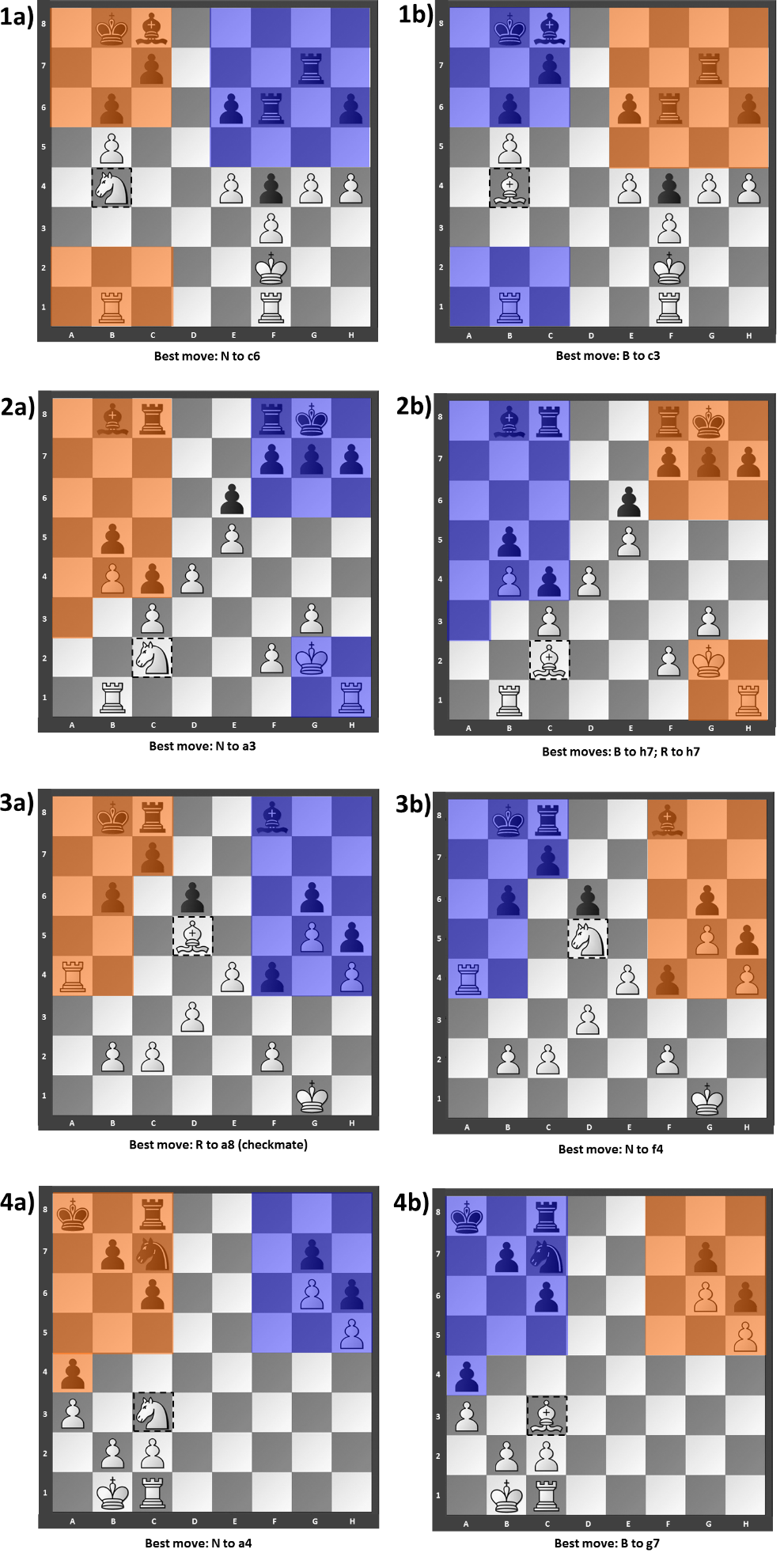


**
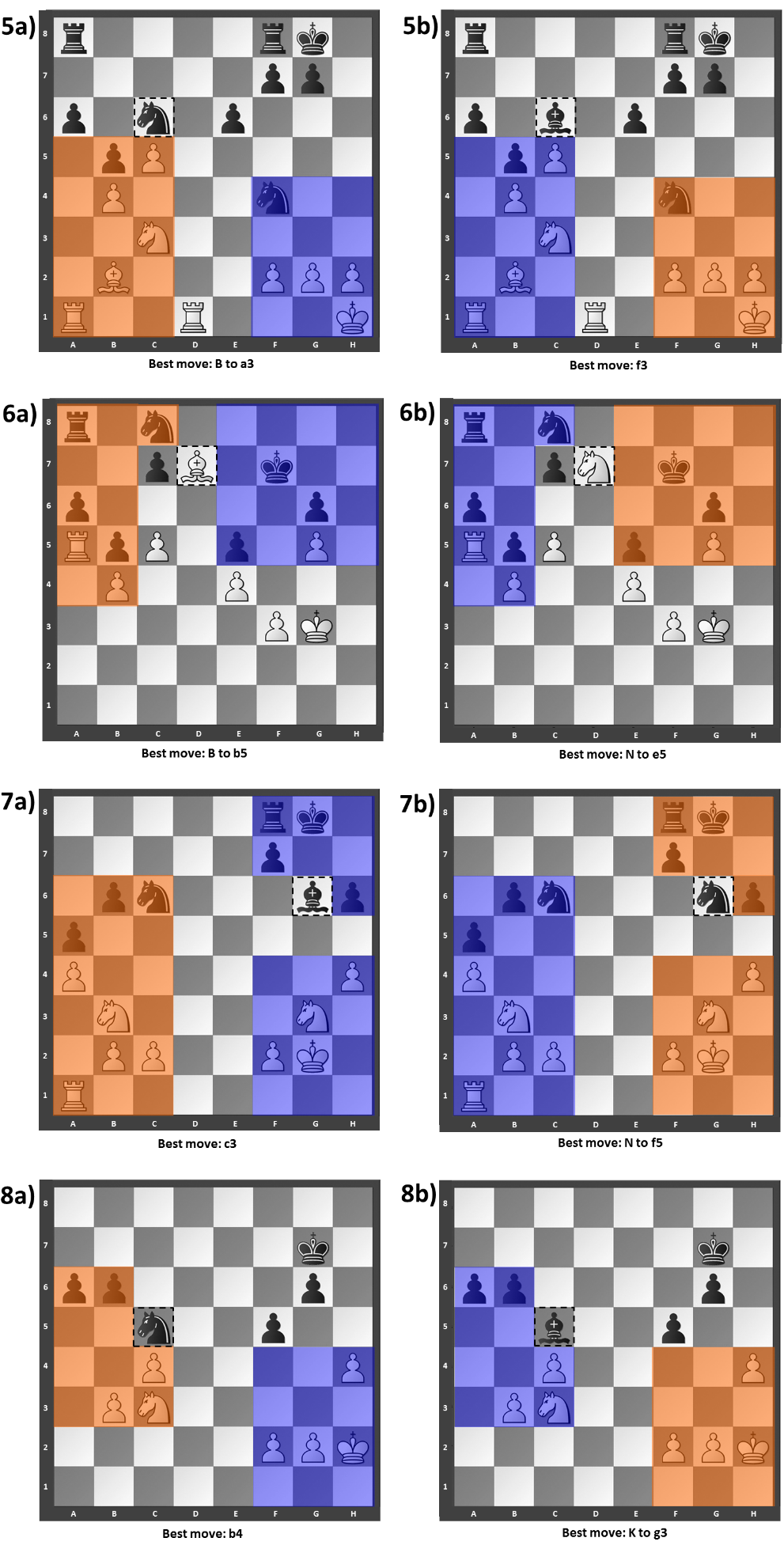
**
